# *De novo* assembly and annotation of *Tetradesmus* sp. strain 198, a green algal from dryland soil

**DOI:** 10.1128/mra.01015-24

**Published:** 2025-04-04

**Authors:** Veronica Malavasi, Núria Beltran-Sanz, Elena Catelan-Carphio, Alessandro Grapputo, David J. Eldridge, Samantha Travers, Fernando T. Maestre, Francesco Dal Grande

**Affiliations:** 1 Botanical Garden, University of Padua 9308, Padua, Italy; 2 Department of Botany, Charles University 580016, Prague, Czechia; 3 Department of Biology, University of Padua 165369, Padua, Italy; 4 National Biodiversity Future Center (NBFC), University of Palermo 28206, Palermo, Italy; 5 Centre for Ecosystem Science, School of Biological, Earth and Environmental Sciences, University of New South Wales 591183 , Sydney, New South Wales, Australia; 6 Conservation and Restoration Science Branch, Department of Climate Change, Energy, the Environment and Water 680196 , Lisarow, Australia; 7 Environmental Sciences and Engineering, Biological and Environmental Science and Engineering Division, King Abdullah University of Science and Technology (KAUST) 564519 , Thuwal, Makkah, Saudi Arabia; University of California Riverside, Riverside, California, USA

**Keywords:** dryland, green algae

## Abstract

*Tetradesmus* sp. strain 198 is a microalga with potential for the biotechnological production of carotenoids. In this study, we have sequenced the genome, obtaining a total contig-level genome assembly length of 149 Mbp. The BUSCO completeness was 91%, the N50 was 783 kbp, and the total number of annotated genes was 19,841.

## ANNOUNCEMENT

*Tetradesmus* sp. strain 198, a newly described strain of the Chlorophyceae class within the Scenedesmaceae family, has been identified as belonging to the *Tetradesmus bajacalifornicus* clade based on reference 1. It was isolated from a bare soil sample in an arid region of Australia’s east coast (34.71686, 144.77065), during the Biodesert global dryland survey (2). The strain was cultivated in an Erlenmeyer flask containing 50 mL of modified Bold Basal Medium (3) under continuous light at 22°C and 100 photons m^−2^ s^−1^ for 2 weeks. DNA extraction was performed using the NucleoSpin Plant II kit (Macherey-Nagel, Düren, Germany) according to the manufacturer’s instructions. Two sequencing libraries were prepared: a Nanopore long-read library, sequenced on the ONT MinION system (15 Gbp of raw reads) and an Illumina short-read library, sequenced on the NovaSeqX Plus platform (6.75 Gbp of raw paired-end reads).

Nanopore reads were base-called using Guppy v.7.1.4 with the fast model (400 bps), R10.4.1 MinION Flow Cells (FLO-MIN114), and the SQK-LSK114 library preparation kit. Reads with a quality score above eight were analyzed with Nanoplot v.1.28.1 (4). The summary statistics generated by Nanoplot indicated a total read count of 3,548,659, with a read length N50 of 5,117 bp. Long reads were then assembled into contig using Flye v.2.9 (5).

Illumina short reads were trimmed with Fastp v.0.23.4 (6), applying base correction, low complexity filtering, and maintaining a minimum read length of 100 bp. The report generated by Fastp shows 41 Mbp of total paired-end reads, with a mean length of 149 bp. Prior to and following the trimming process, the quality of the Illumina raw reads was evaluated using FASTQC v.0.11.9 (7). The trimmed Illumina reads were then mapped to the contig-level assembly using BWA-MEM v.0.7.17 (8), and the base-level accuracy was further improved by an iteration of short-read polishing using Pilon v.1.24 (9). We further evaluated potential contamination with Blobtools v.1.1.1 (10) utilizing the generated mapping files and a BLASTN v.2.14.0 (11) against the NCBI nt database (https://www.ncbi.nlm.nih.gov/). Contamination and organelles were then extracted from the main assembly.

**Fig 1:**
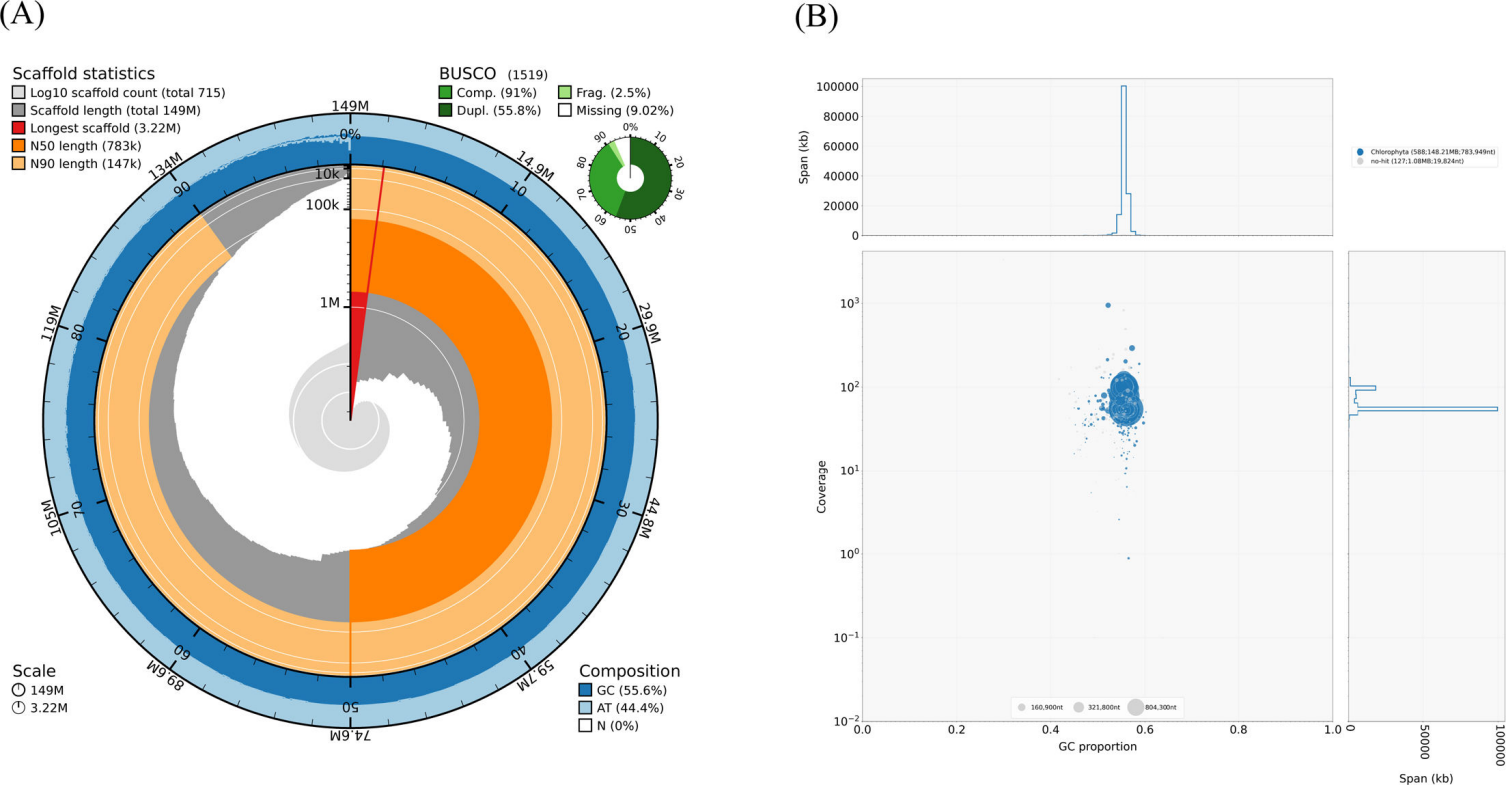
(**A**) SnailPlot and (**B**) BlobPlot analysis of *Tetradesmus* sp. strain 198 genome.

The summary of the main characteristics of the genome assembly is shown in the Snailplot performed with Blobtoolkit v.4.2.1 (12, see Fig. 1). The final assembly consists of 715 contigs, with a total length of 149 Mbp, an N50 of 783 kbp, a guanine-cytosine (GC) content of 55.56%, and a BUSCO completeness of 91%. Coverage was evaluated with QualiMap v.2.3 (13), resulting in 30× and 89× for long and short reads, respectively.

Masking of the genome with RepeatModeler v.2.0.5 (14) and RepeatMasker v.4.1.0 (15) showed a repeat content of 19%. Gene annotation identified 19,841 genes using GeMoMa v.9 (16), based on seven Chlorophyta reference genomes from NCBI RefSeq.

Default parameters were used except where otherwise noted.

## Data Availability

The genome assembly has been deposited in NCBI under BioProject accession GCA_043380735.1 and BioSample SAMN42569494. The Genbank accession number is JBIEIR000000000. Nanopore raw reads are available under SRR30668593 and Illumina raw reads under SRR32050663. The GeMoMa annotation file is accessible on Zenodo (https://doi.org/10.5281/zenodo.14696946).
